# Comparison of clinical outcomes after drug-eluting balloon and drug-eluting stent use for in-stent restenosis related acute myocardial infarction: a retrospective study

**DOI:** 10.7717/peerj.4646

**Published:** 2018-04-18

**Authors:** Chih-Yuan Fang, Hsiu-Yu Fang, Chien-Jen Chen, Cheng-Hsu Yang, Chiung-Jen Wu, Wei-Chieh Lee

**Affiliations:** Division of Cardiology, Department of Internal Medicine, Kaohsiung Chang Gung Memorial Hospital, Chang Gung University College of Medicine, Kaohsiung, Taiwan

**Keywords:** Drug-eluting stent, Acute myocardial infarction, Drug-eluting balloon, In-stent restenosis

## Abstract

**Background:**

Good results of drug-eluting balloon (DEB) use are achieved in in-stent restenosis (ISR) lesions, small vessel disease, long lesions, and bifurcations. However, few reports exist about DEB use in acute myocardial infarction (AMI) with ISR. This study’s aim was to evaluate the efficacy of DEB for AMI with ISR.

**Methods:**

Between November 2011 and December 2015, 117 consecutive patients experienced AMI including ST-segment elevation MI, and non-ST-segment elevation MI due to ISR, and received percutaneous coronary intervention (PCI). We divided our patients into two groups: (1) PCI with further DEB, and (2) PCI with further drug-eluting stent (DES). Clinical outcomes such as target lesion revascularization, target vessel revascularization, recurrent MI, stroke, cardiovascular mortality, and all-cause mortality were analyzed.

**Results:**

The patients’ average age was 68.37 ± 11.41 years; 69.2% were male. A total of 75 patients were enrolled in the DEB group, and 42 patients were enrolled in the DES group. The baseline characteristics between the two groups were the same without statistical differences except for gender. Peak levels of cardiac biomarker, pre- and post-PCI cardiac function were similar between two groups. The major adverse cardiac cerebral events rate (34.0% vs. 35.7%; *p* = 0.688) and cardiovascular mortality rate (11.7% vs. 12.8%; *p* = 1.000) were similar in both groups.

**Conclusions:**

DEB is a reasonable strategy for AMI with ISR. Compared with DES, DEB is an alternative strategy which yielded acceptable short-term outcomes and similar 1-year clinical outcomes.

## Introduction

With the improvement of the technology and the design of the stent, the incidence of in-stent restenosis (ISR) decreased. Drug-eluting balloons (DEBs) have become an important alternative to the current treatment of ISR (Wöhrle et al., 2012). The 2014 European guideline for coronary revascularization recommends the use of DEB for the treatment of ISR of a bare-metal stent (BMS) or drug-eluting stent (DES) (Class I, level of evidence: B) (Windecker et al., 2014). However, the American College of Cardiology/American Heart Association/Society for Cardiovascular Intervention guidelines for percutaneous coronary intervention (PCI) recommends DES to treat BMS ISR (Class I, Level of Evidence: A) and plain old balloon angioplasty, BMS, or DES to treat DES-ISR (Levine et al., 2011). The guideline does not issue any recommendations for DEB. In real-world practice, the use of DEB for either BMS or DES restenosis showed good clinical results (Stella et al., 2011; Lee et al., 2016). DEBs provide advantages over DESs, such as rapid release of drug to the arterial wall, the absence of polymers and stent structures, and the absence of stent thrombosis (Waksman & Pakala, 2009). Paclitaxel has been identified as the primary drug for use in DEBs because of its long-lasting antiproliferative effect and retained uptake by vascular smooth muscle cells up to one week (Waksman & Pakala, 2009). On the other hand, DEB also has been applied for *de novo* coronary lesions, small vessel disease, long lesions, and bifurcations, and presented good results (Fröhlich et al., 2013; Vaquerizo et al., 2015; Richelsen, Overvad & Jensen, 2016).

However, there are few data about use of DEB in acute coronary syndromes, especially acute myocardial infarction (AMI). One DEB-AMI trial (Belkacemi et al., 2012) in ST-segment elevation myocardial infarction (STEMI) patients, angiographic results of DES were superior to both BMS and DEB plus BMS. However, no clinical outcomes of DEB for STEMI and no other randomized trials about DEB use in non-ST-segment elevation MI (NSTEMI) were achieved. Most trials and real-world practice also use DEB for relative stable conditions such as stable angina and unstable angina (Wöhrle et al., 2012; Lee et al., 2016; Fröhlich et al., 2013).

Therefore, we focused on the use of DEB for acute conditions with thrombus. This study’s aim was to compare the differences in clinical outcomes between the use of DES and DEB for AMI with ISR.

## Materials and Methods

The study was approved by the Institutional Review Committee on Human Research of Chang Gung Memorial Hospital for retrospective analysis in consecutive patients with AMI including STEMI and NSTEMI who underwent PCI with DEB and DES for ISR between November 2011 and December 2015 in our hospital. The approval number was 201701790B0. The raw data was from the myocardial infarction registry of Kaohsiung Chang Gung Memorial Hospital.

### Patients and groups

Between November 2011 and December 2015, 117 consecutive patients with AMI and received PCI for ISR were retrospectively enrolled. All patients refused CABG due to high operation risk and patients’ choice. The patients were divided into two groups: (1) PCI with further DEB, and (2) PCI with further DES. In both groups, dual anti-platelet therapy (Aspirin plus clopidogrel or ticagrelor) was used for one year for AMI. The decision of using DEB or DES was on the operator’s discretion. The most reasons that the operator choose DEB were: (1) the lesions had more than two layers of metallic stents; (2) the lesions were relatively less of a plaque burden after balloon angioplasty.

### Endpoints

Clinical outcomes such as target lesion revascularization (TLR), target vessel revascularization (TVR), recurrent MI, stroke, cardiovascular mortality, and all-cause mortality were analyzed. In-hospital major adverse cardiac cerebral events (MACCEs), cardiovascular mortality, and all-cause mortality were compared between the two groups.

### Definitions

AMI definitions were in accordance with the most recent universal definition of AMI (Thygesen et al., 2012). TLR was defined as a repeat PCI or CABG for a lesion in the previously treated segment or in an adjacent 5 mm segment. TVR was defined as a repeat PCI in a target vessel. MACCEs included TLR, TVR, recurrent MI, stroke, and cardiovascular mortality. Cardiovascular mortality was defined as death related to cardiac arrhythmia, heart failure, and cardiogenic shock. All-cause mortality was defined as death from any cause.

### Study endpoints

The primary endpoints were a MACCE during the follow-up period. The secondary endpoint was all-cause mortality during the follow-up period.

### Statistical analysis

Data are expressed as a mean ± standard deviation for continuous variables or as counts and percentages for categorical variables. Continuous variables were compared using an independent sample t or Mann–Whitney *U* tests. Categorical variables were compared using a Chi-square statistic. A Kaplan–Meier curve was performed with log rank test for TLR, TVR, recurrent MI, and cardiovascular mortality in DEB and DES groups during the 1-year follow-up period.

Because the patients were not randomly assigned, there was some bias in baseline characteristics. In order to compare the clinical effect between DEB and DES, a propensity score matched analysis was performed as a 1-to-1 matched analysis using a logistic regression model for the DEB group versus the DES group to adjust for differences in baseline characteristics. Using the estimated logits, the DEB group and the DES group had the closest estimated logit value. The baseline covariates were compared between these two groups and were similar.

All statistical analyses were performed using SPSS 22.0 (IBM Corp., Armonk, NY, USA). A *p*-value <0.05 was considered statistically significant.

**Table 1:** Patient characteristics of DEB and DES group.

	DEB (*N* = 75; *L* = 103)	DES (*N* = 42; *L* = 54)	*P* value
***General demographics***			
Age (years)	67.53 ± 11.62	69.86 ± 11.00	0.292
Male sex (%)	46 (61.3)	35 (83.3)	0.021
***Indication***			0.541
STEMI (%)	7 (9.3)	6 (14.3)	
NSTEMI (%)	68 (90.7)	36 (85.7)	
***Killip classification***			0.079
I (%)	34 (45.3)	29 (69.0)	
II (%)	13 (17.3)	4 (9.5)	
III (%)	17 (22.7)	7 (16.7)	
IV (%)	11 (14.7)	2 (4.8)	
***Risk factors for MI***			
Diabetes (%)	58 (77.3)	26 (61.9)	0.089
Current smoker (%)	25 (33.3)	16 (38.1)	0.687
Hypertension (%)	64 (85.3)	37 (88.1)	0.784
Prior MI (%)	32 (42.7)	15 (35.7)	0.556
Prior stroke (%)	8 (10.7)	7 (16.7)	0.394
PAOD (%)	16 (21.3)	4 (9.5)	0.129
Dyslipidemia (%)	39 (52.0)	23 (54.8)	0.848
CABG (%)	5 (6.7)	3 (7.1)	1.000
ESRD on maintenance hemodialysis (%)	28 (37.3)	12 (28.6)	0.418
Heart failure (%)	34 (45.3)	19 (45.2)	1.000
***Laboratory examination***			
Creatinine (except ESRD) (mg/dL)	2.08 ± 1.09	1.46 ± 0.82	0.132
CK-MB (ng/mL)	51.57 ± 11.84	76.59 ± 15.95	0.166
Troponin-I (ng/mL)	18.07 ± 8.59	24.72 ± 10.86	0.244
***Left ventricular ejection fraction (%)***			
Before	55.47 ± 12.06	51.42 ± 12.79	0.117
After	59.26 ± 11.75	55.13 ± 13.08	0.182
***Characteristics of coronary artery disease***			
Single or multiple-vessel disease			0.252
Single vessel disease (%)	3 (4.0)	4 (9.5)	
Double vessel disease (%)	16 (21.3)	5 (11.9)	
Triple vessel disease (%)	56 (74.7)	33 (78.6)	
Left main disease (%)	23 (30.7)	12 (28.6)	0.837
***Previous stent***			0.867
Bare-metal stent (%)	59 (57.3)	30 (55.6)	
Drug-eluting stent (%)	44 (42.7)	24 (44.4)	
***Infarcted artery***			0.174
Left main (%)	1 (1.0)	3 (5.6)	
Left anterior descending artery (%)	46 (44.7)	21 (38.9)	
Left circumflex artery (%)	25 (24.3)	9 (16.7)	
Right coronary artery (%)	31 (30.1)	21 (38.9)	
***One-year follow-up angiography (%)***	34 (45.3)	23 (54.8)	0.343

**Notes.**

Data are expressed as mean ± SD or as number (percentage).

*N* number *L* lesion DEB drug-eluting balloon DES drug-eluting stent STEMI ST-segment elevation myocardial infarction NSTEMI non ST-segment elevation myocardial infarction MI myocardial infarction PAOD peripheral arterial occlusive disease CABG coronary artery bypass grafting ESRD end stage renal disease CK-MB creatine kinase-MB

## Results

### Patient characteristics (Table 1)

The average age of the patients in both groups was similar, but the percentage of males was lower in the DEB group (61.3% vs. 83.3%; *p* = 0.021). Most patients presented NSTEMI in the DEB and DES groups (90.7% vs. 83.3%), and most patients presented Killip I status. The risk factors for AMI were similar between the two groups. The level of serum creatinine was similar in the both groups (2.08 ± 1.09 mg/dL vs. 1.46 ± 0.82 mg/dL; *p* = 0.132). Similar prevalence of multiple vessel coronary artery disease (96.0% vs. 90.5%) and left main coronary artery disease (30.7% vs. 28.6%) was observed in both groups. The previous stents and the infarcted artery were similar between groups. The prevalence of follow-up coronary angiography was 45.3% in the DEB group and 54.8% in the DES group (*p* = 0.343).

### Lesion characteristics (Table 2)

Most lesions were diffuse (63.1% vs. 59.3%) in both groups. Greater pre-PCI stenotic percentage (82.84 ± 12.33% vs. 78.55 ± 8.99%; *p* = 0.025) and greater post-PCI stenotic percentage (16.68 ± 8.88% vs. 12.03 ± 6.10%; *p* = 0.001) were seen in the DEB group. Lower post-PCI minimal luminal diameter (MLD) (2.50 ± 0.55 mm vs. 2.89 ± 0.56 mm; *p* < 0.001) and post-PCI reference luminal diameter (RLD) (3.05 ± 0.65 mm vs. 3.29 ± 0.60 mm; *p* = 0.028) were seen in the DEB group. The percentage of intravascular ultrasound study (IVUS) use was similar in both groups (32.0% vs. 38.9%; *p* = 0.480). Smaller diameter of the size of DEB (3.08 ± 0.42 mm vs. 3.23 ± 0.43 mm; *p* = 0.042) and similar length of the DEB (26.41 ± 4.20 mm vs. 28.02 ± 7.81 mm; *p* = 0.095) were noted when comparing with the size of DES.

**Table 2:** Lesion characteristics of DEB and DES group.

	DEB (*N* = 75; *L* = 103)	DES (*N* = 42; *L* = 54)	*P* value
***Lesion type***			0.730
Focal lesion (%)	38 (36.9)	22 (40.7)	
Diffuse lesion (%)	65 (63.1)	32 (59.3)	
***The characteristics of lesion***			
Pre-PCI stenosis (%)	82.84 ± 12.33	78.55 ± 8.99	0.025
MLD (mm)	0.52 ± 0.40	0.60 ± 0.30	0.185
RLD (mm)	2.93 ± 0.65	2.88 ± 0.63	0.668
Post-PCI stenosis (%)	16.68 ± 8.88	12.03 ± 6.10	0.001
MLD (mm)	2.50 ± 0.55	2.89 ± 0.56	<0.001
RLD (mm)	3.05 ± 0.65	3.29 ± 0.60	0.028
***The use of intravascular ultrasound study (%)***	33 (32.0)	21 (38.9)	0.480
***The characteristics of DEB or DES***			
Diameter (mm)	3.08 ± 0.42	3.23 ± 0.43	0.042
Length (mm)	26.41 ± 4.20	28.02 ± 7.81	0.095

**Notes.**

Data are expressed as mean ± SD or as number (percentage).

*N* number *L* lesion DEB drug-eluting balloon DES drug-eluting stent PCI percutaneous coronary intervention MLD minimal luminal diameter RLD reference luminal diameter

### One-year clinical outcomes of DEB and DES group (Table 3)

Between two groups, the incidence of events including in-hospital MACCE, total MACCE, TLR, TVR, recurrent MI, stroke, cardiovascular death, and all-cause mortality were similar.

**Table 3:** One-year clinical outcomes of DEB and DES group.

	DEB (*N* = 75; *L* = 103)	DES (*N* = 42; *L* = 54)	*P* value
In-hospital MACCE (%)	2 (2.7)	3 (7.1)	0.348
MACCE (%)	24 (34.0)	15 (35.7)	0.688
Target-lesion revascularization (%)	21 (26.9)	9 (22.5)	0.661
Target-vessel revascularization (%)	25 (31.3)	11 (27.5)	0.833
Recurrent myocardial infarction (%)	9 (16.1)	7 (21.9)	0.570
STEMI (%)	0	1 (14.3)	
NSTEMI (%)	9 (100)	6 (85.7)	
Stroke (%)	2 (3.6)	1 (3.1)	1.000
Cardiovascular mortality (%)	7 (11.7)	5 (12.8)	1.000
All-cause mortality (%)	16 (22.9)	7 (17.1)	0.628

**Notes.**

Data are expressed as mean ± SD or as number (percentage).

*N* number *L* lesion DEB drug-eluting balloon DES drug-eluting stent MACCE major adverse cardiac cerebral event STEMI ST-segment elevation myocardial infarction NSTEMI non ST-segment elevation myocardial infarction

### The Kaplan–Meier curves of 1-year clinical outcomes of DEB and DES groups in TLR, TVR, recurrent MI, and cardiovascular mortality (Fig. 1)

**Figure 1:**
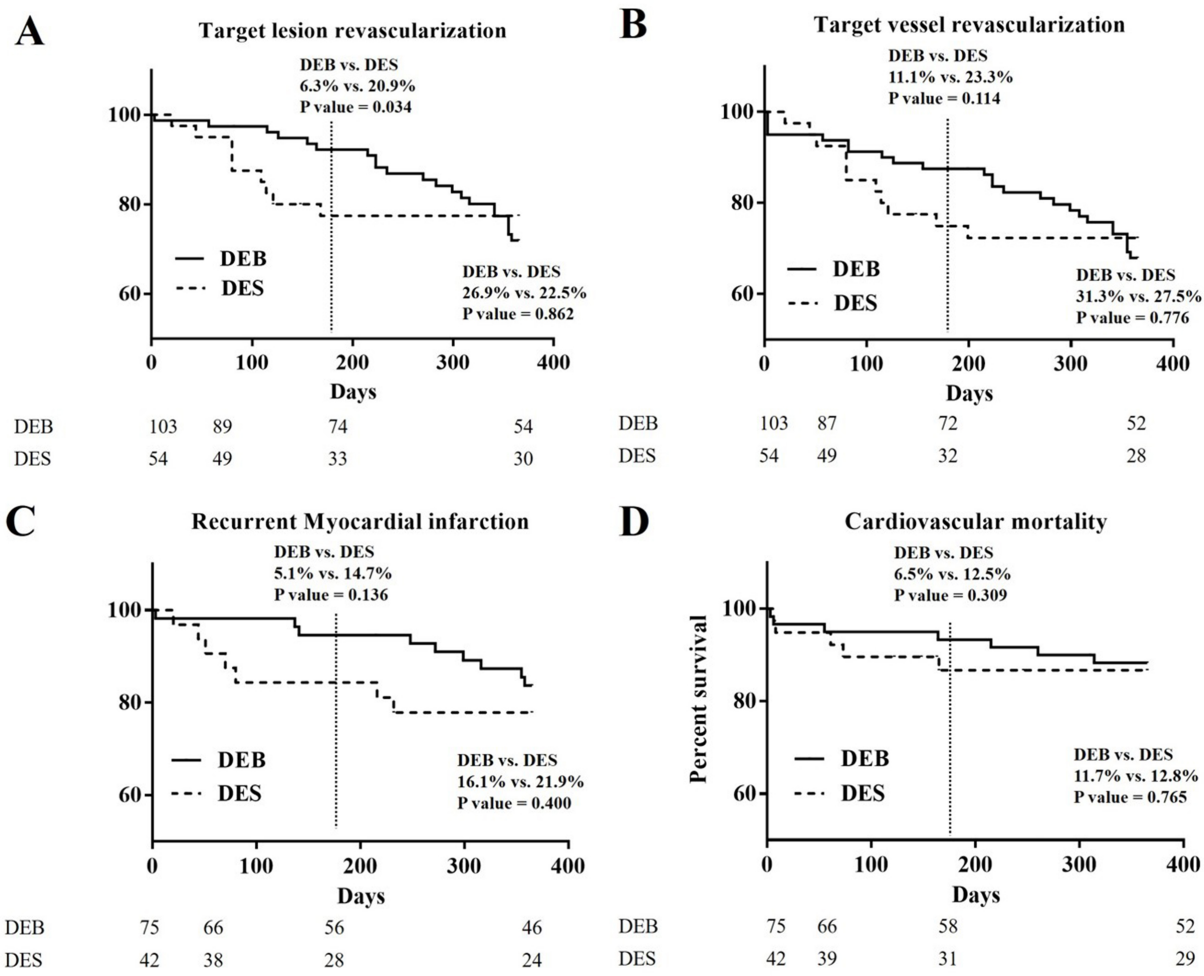
The Kaplan–Meier curves of 1-year clinical outcomes of DEB and DES group in TLR, TVR, recurrent MI, and cardiovascular mortality. (A) The Kaplan–Meier curve of 1-year TLR: significant difference at the half-year follow-up period (6.3% vs. 20.9%; *p* = 0.034) was noted. No difference at 1-year follow-up period (26.9% vs. 22.5%; *p* = 0.862) was noted. (B) The Kaplan-Meier curve of 1-year TVR: no significant difference was noted at the half-year follow-up period (11.1% vs. 23.3%; *p* = 0.114), and at the 1-year follow-up period (31.3% vs. 27.5%; *p* = 0.776). (C) The Kaplan-Meier curve of 1-year recurrent myocardial infarction: no significant difference was noted at at the half-year follow-up period (5.1% vs. 14.7%; *p* = 0.136), and at 1-year follow-up period (16.1% vs. 21.9%; *p* = 0.400). (D) The Kaplan-Meier curve of 1-year cardiovascular mortality: The Kaplan-Meier curve of 1-year TVR: no significant difference was noted at the half-year follow-up period (6.5% vs. 12.5%; *p* = 0.309), and at 1-year follow-up period (11.7% vs. 12.8%; *p* = 0.765).

In Fig. 1A, the Kaplan–Meier curve of 1-year TLR showed significant difference at the half-year follow-up period (6.3% vs. 20.9%; *p* = 0.034), and became no different at the 1-year follow-up period (26.9% vs. 22.5%; *p* = 0.862). In Fig. 1B, the Kaplan–Meier curve of 1-year TVR showed no significant difference at the half-year follow-up period (11.1% vs. 23.3%; *p* = 0.114), and at 1-year follow-up period (31.3% vs. 27.5%; *p* = 0.776). In Fig. 1C, the Kaplan–Meier curve of recurrent MI showed no significant difference at the half-year follow-up period (5.1% vs. 14.7%; *p* = 0.136), and at the 1-year follow-up period (16.1% vs. 21.9%; *p* = 0.400). In Fig. 1D, the Kaplan–Meier curve of cardiovascular death showed no significant difference at the half-year follow-up period (6.5% vs. 12.5%; *p* = 0.309), and at the 1-year follow-up period (11.7% vs. 12.8%; *p* = 0.765).

### Patient and lesion characteristics, and one-year clinical outcomes of DEB and DES group after propensity score matched (Tables 4 and 5)

After propensity score matched, all baseline characteristic became similar between two groups except post-PCI MLD. In addition, higher incidence of TLR (4.9% vs. 22.0%; *p* = 0.048) and TVR (4.9% vs. 24.9%; *p* = 0.026) was noted at the half-year follow-up period.

**Table 4:** Patient and lesion characteristics of DEB and DES group after propensity score matched.

	DEB (*N* = 40; *L* = 52)	DES (*N* = 40; *L* = 52)	*P* value
***General demographics***			
Age (years)	67.78 ± 13.04	69.48 ± 11.00	0.530
Male sex (%)	34 (85.0)	33 (82.5)	1.000
***Indication***		0.481	
STEMI (%)	3 (7.5)	6 (15.0)	
NSTEMI (%)	37 (92.5)	34 (85.0)	
***Killip classification***			
≥III (%)	10 (25.0)	9 (22.5)	1.000
***Risk factors for MI***			
Diabetes (%)	24 (60.0)	26 (65.0)	0.818
Current smoker (%)	20 (50.0)	15 (37.5)	0.367
Hypertension (%)	35 (87.5)	35 (87.5)	1.000
Prior MI (%)	15 (37.5)	13 (32.5)	0.815
Prior stroke (%)	5 (12.5)	7 (17.5)	0.755
PAOD (%)	6 (15.0)	4 (10.0)	0.737
Dyslipidemia (%)	24 (60.0)	23 (57.5)	1.000
CABG (%)	4 (10.0)	3 (7.5)	1.000
ESRD on maintenance hemodialysis (%)	13 (32.5)	12 (30.0)	1.000
Heart failure (%)	14 (30.0)	18 (45.0)	0.494
***Laboratory examination***			
CK-MB (ng/mL)	58.42 ± 17.24	68.86 ± 19.71	0.638
Troponin-I (ng/mL)	17.92 ± 8.91	22.45 ± 9.75	0.492
***Left ventricular ejection fraction (%)***			
Before	53.59 ± 11.96	50.95 ± 13.93	0.059
After	58.94 ± 11.84	55.72 ± 13.19	0.137
***Characteristics of coronary artery disease***			
Multiple-vessel disease	30 (75.0)	32 (80.0)	0.620
Left main disease (%)	13 (32.5)	12 (30.0)	1.000
***Previous stent***			1.000
Bare-metal stent (%)	30 (57.7)	30 (57.7)	
Drug-eluting stent (%)	22 (42.3)	22 (42.3)	
***Infarcted artery***			0.330
Left main (%)	0 (0)	3 (5.8)	
Left anterior descending artery (%)	20 (38.5)	20 (38.5)	
Left circumflex artery (%)	12 (23.1)	9 (17.3)	
Right coronary artery (%)	20 (38.5)	20 (38.5)	
***Lesion type***			0.692
Focal lesion (%)	24 (46.2)	21 (40.4)	
Diffuse lesion (%)	28 (53.8)	31 (59.6)	
***The characteristics of lesion***			
Pre-PCI stenosis (%)	81.50 ± 12.75	78.65 ± 9.11	0.193
MLD (mm)	0.59 ± 0.43	0.59 ± 0.30	0.975
RLD (mm)	2.95 ± 0.60	2.88 ± 0.64	0.553
Post-PCI stenosis (%)	14.83 ± 8.04	12.07 ± 6.19	0.052
MLD (mm)	2.64 ± 0.59	2.89 ± 0.57	0.032
RLD (mm)	3.10 ± 0.69	3.29 ± 0.61	0.140
***The use of intravascular ultrasound study (%)***	13 (32.5)	15 (37.5)	0.815
***The characteristics of DEB or DES***			
Diameter (mm)	3.16 ± 0.43	3.23 ± 0.44	0.462
Length (mm)	26.50 ± 4.21	27.64 ± 7.70	0.353

**Notes.**

Data are expressed as mean ± SD or as number (percentage).

*N* number *L* lesion DEB drug-eluting balloon DES drug-eluting stent STEMI ST-segment elevation myocardial infarction NSTEMI non ST-segment elevation myocardial infarction MI myocardial infarction PAOD peripheral arterial occlusive disease CABG coronary artery bypass grafting ESRD end stage renal disease CK-MB creatine kinase-MB PCI percutaneous coronary intervention MLD minimal luminal diameter RLD reference luminal diameter

**Table 5:** One-year clinical outcomes of DEB and DES group after propensity score matched.

	DEB (*N* = 40; *L* = 52)	DES (*N* = 40; *L* = 52)	*P* value
In-hospital MACCE (%)	0 (0)	3 (7.5)	0.241
***Half-year***			
Target-lesion revascularization (%)	2 (4.9)	9 (22.0)	0.048
Target-vessel revascularization (%)	2 (4.9)	10 (24.4)	0.026
***One-year***			
MACCE (%)	11 (27.5)	15 (37.5)	0.474
Target-lesion revascularization (%)	14 (34.1)	9 (23.7)	0.333
Target-vessel revascularization (%)	14 (34.1)	11 (28.9)	0.638
Recurrent myocardial infarction (%)	3 (10.0)	7 (23.3)	0.299
Stroke (%)	1 (3.2)	1 (3.3)	1.000
Cardiovascular mortality (%)	0 (0)	5 (13.5)	0.060
All-cause mortality (%)	6 (16.7)	7 (17.9)	1.000

**Notes.**

Data are expressed as mean ± SD or as number (percentage).

*N* number *L* lesion DEB drug-eluting balloon DES drug-eluting stent MACCE major adverse cardiac cerebral event

## Discussion

In the present study, the baseline characteristics were similar, expect for gender. Most patients had multiple vessel coronary artery disease and had undergone BMS implantation previously. Most ISR were diffuse lesions in both groups. DEB group had worse pre-PCI stenotic percentage and worse post-PCI results than the DES group. These results were related to no metallic structure in the DEB group, but did not influence clinical outcomes. The DEB group experienced less recurrent MI during the half-year and 1-year follow-up periods due to no metallic structure. In the DEB group, better results at about half-year TLR and half-year TVR were noted, but similar results at about 1-year TLR and TVR were noted. A relatively lower percentage of IVUS use was related to an emergent condition. According to our results, use of DEB for ISR with AMI had similar results as use of DES, and could decrease the possibilities of short-term events.

Paclitaxel is the most effective drug used with DEB technology due to its significant lipophilia, which allows for a more homogeneous distribution through the vessel wall, as well as a quick absorption and the duration of the effect, which may be extended for several days (Waksman & Pakala, 2009). Many studies including randomized controlled trials and meta-analyses showed good results of DEB for ISR compared with conventional balloon angioplasty (Habara et al., 2011; Indermuehle et al., 2013), and similar results of DEB for ISR compared with DES (Alfonso et al., 2014b). Most patients presented a relatively stable condition such as silent ischemia, stable angina, and unstable angina in randomized controlled trials (Habara et al., 2011; Indermuehle et al., 2013; Alfonso et al., 2014b). However, there are few data about DEB in acute coronary syndromes, especially AMI. Compared with BMS alone for NSTEMI, patients treated with BMS plus DEB had significantly less luminal loss, but the treatment did not affect patient clinical outcomes (Besic et al., 2015). In STEMI, angiographic results of DES were superior to both BMS and DEB plus BMS (Belkacemi et al., 2012). Therefore, physicians preferred to use the metallic stent first for AMI due to high thrombus condition, and used the DEB to mimic DES. Currently, no head-to-head randomized study has been performed to compare the clinical outcome between only DEB and DES for AMI.

DES improved the outcome of AMI after PCI, but recurrent ISR and stent thrombosis still were difficult problems. “Stent in stent” treatment increases the possibilities of stent thrombosis due to luminal loss, chronic inflammation and hypersensitivity reactions (Alfonso et al., 2014a). Repeat stenting for ISR may be related to insufficient stent expansion and suboptimal stent geometry because restenotic or thrombosed stents are difficult to reopen (Seedial et al., 2013). DEBs have recently had a potential to overcome the limitations of DESs. Some limitations of DESs are the need for long lengths to cover the entire surface of a diseased vessel, their association with excessive intimal hyperplasia, and difficulties about adaptive remodeling of restenosis (Seedial et al., 2013). In the present study, short-term data also showed higher incidence of TLR, TVR, and recurrent MI in the DES group. Therefore, DEBs have emerged as a potential alternative to the current treatment of ISR, and provide the freedom of polymers and stent structures. Therefore, DEBs prevent the problem of “stent in stent”, do not cause stent thrombosis and luminal loss, and may decrease the possibility of sudden death if acute stent thrombosis happens after repeat stenting. In addition, few have reported about DEB use for combined ISR and acute coronary syndrome, especially AMI with high thrombus contained condition.

In the present study, the patients in both groups had similar baseline characteristics, even though non-randomized controlled study. Use of DEBs seems to provide good short-term outcomes and less TLR, TVR, and recurrent MI due to no stent thrombosis. However, the clinical outcome became similar at 1-year follow-up period. In both groups, a relatively higher event rate was noted, because the study population experienced recurrent ISR and had multiple comorbidities including diabetes, and ESRD, and multiple vessel coronary artery disease. CABG could be an option for the patient experienced recurrent ISR and had multiple vessel coronary artery disease. In this study, all patients refused CABG due to high operation risk and patients’ choice. In this study, we focused on the impact of DEB for the combination of AMI and ISR in clinical practice for a high risk population.

### Limitations

The present study had some limitations, including being a non-randomized study and having selection bias because the operator may consider the use of DES in the patients with complex lesions and the use of DEB in the patients with shorter and simple lesions. Even though the present study is non-randomized, the baseline characteristics were very similar between the two groups. In addition, no previous study compared clinical outcomes between only DEB and DES use for AMI with ISR. Our study provided the insight on the use of DEB for AMI in clinical practice for high risk population.

## Conclusions

DEB is a reasonable strategy for AMI with ISR. Compared with DES, DEB was an alternative strategy which yielded acceptable short-term outcomes and similar 1-year clinical outcomes.

##  Supplemental Information

10.7717/peerj.4646/supp-1 Data S1 DEB for MI raw data

## Additional Information and Declarations

### Data Availability

The following information was supplied regarding data availability: The raw data are provided in Data S1.

## References

[ref-1] Alfonso F, Byrne RA, Rivero F, Kastrati A (2014a). Current treatment of in-stent restenosis. Journal of the American College of Cardiology.

[ref-2] Alfonso F, Pérez-Vizcayno MJ, Cárdenas A, García Del Blanco B, Seidelberger B, Iñiguez A, Gómez-Recio M, Masotti M, Velázquez MT, Sanchís J, García-Touchard A, Zueco J, Bethencourt A, Melgares R, Cequier A, Dominguez A, Mainar V, López-Mínguez JR, Moreu J, Martí V, Moreno R, Jiménez-Quevedo P, Gonzalo N, Fernández C (2014b). A randomized comparison of drug-eluting balloon versus everolimus-eluting stent in patients with bare-metal stent-in-stent restenosis: the RIBS V Clinical Trial (Restenosis Intra-stent of Bare Metal Stents: paclitaxel-eluting balloon vs. everolimus-eluting stent). Journal of the American College of Cardiology.

[ref-3] Windecker S, Kolh P, Alfonso F, Collet JP, Cremer J, Falk V, Filippatos G, Hamm C, Head SJ, Jüni P, Kappetein AP, Kastrati A, Knuuti J, Landmesser U, Laufer G, Neumann FJ, Richter DJ, Schauerte P, Sousa Uva M, Stefanini GG, Taggart DP, Torracca L, Valgimigli M, Wijns W, Witkowski A, Authors/Task Force members (2014). ESC/EACTS Guidelines on myocardial revascularization: the task force on myocardial revascularization of the European Society of Cardiology (ESC) and the European Association for Cardio-Thoracic Surgery (EACTS) developed with the special contribution of the European Association of Percutaneous Cardiovascular Interventions (EAPCI). European Heart Journal.

[ref-4] Belkacemi A, Agostoni P, Nathoe HM, Voskuil M, Shao C, Van Belle E, Wildbergh T, Politi L, Doevendans PA, Sangiorgi GM, Stella PR (2012). First results of the DEBAMI (drug eluting balloon in acute ST-segment elevation myocardial infarction) trial: a multicentre randomized comparison of drug-eluting balloon plus bare-metal stent versus bare-metal stent versus drug-eluting stent in primary percutaneous coronary intervention with 6-month angiographic, intravascular, functional, and clinical outcomes. Journal of the American College of Cardiology.

[ref-5] Besic KM, Strozzi M, Margetic E, Bulum J, Kolaric B (2015). Drug eluting balloons in patients with non-ST elevation acute coronary syndrome. Journal of Cardiology.

[ref-6] Fröhlich GM, Lansky AJ, Ko DT, Archangelidi O, De Palma R, Timmis A, Meier P (2013). Drug eluting balloons for *de novo* coronary lesions—a systematic review and meta-analysis. BMC Medicine.

[ref-7] Habara S, Mitsudo K, Kadota K, Goto T, Fujii S, Yamamoto H, Katoh H, Oka N, Fuku Y, Hosogi S, Hirono A, Maruo T, Tanaka H, Shigemoto Y, Hasegawa D, Tasaka H, Kusunose M, Otsuru S, Okamoto Y, Saito N, Tsujimoto Y, Eguchi H, Miyake K, Yoshino M (2011). Effectiveness of paclitaxel-eluting balloon catheter in patients with sirolimus-eluting stent restenosis. Journal of the American College of Cardiology Cardiovascular Intervention.

[ref-8] Indermuehle A, Bahl R, Lansky AJ, Froehlich GM, Knapp G, Timmis A, Meier P (2013). Drug-eluting balloon angioplasty for in-stent restenosis: a systematic review and meta-analysis of randomized controlled trials. Heart.

[ref-9] Lee WC, Fang YN, Fang CY, Chen CJ, Yang CH, Yip HK, Hang CL, Wu CJ, Fang HY (2016). Comparison of clinical results following the use of drug-eluting balloons for a bare-metal stent and drug-eluting stent instent restenosis. Journal of Interventional Cardiology.

[ref-10] Levine GN, Bates ER, Blankenship JC, Bailey SR, Bittl JA, Cercek B, Chambers CE, Ellis SG, Guyton RA, Hollenberg SM, Khot UN, Lange RA, Mauri L, Mehran R, Moussa ID, Mukherjee D, Nallamothu BK, Ting HH (2011). 2011 ACCF/AHA/SCAI Guideline for Percutaneous Coronary Intervention: executive summary. A report of the American College of Cardiology Foundation/American Heart Association Task Force on Practice Guidelines and the Society for Cardiovascular Angiography and Interventions. Circulation.

[ref-11] Richelsen RK, Overvad TF23, Jensen SE (2016). Drug-Eluting balloons in the treatment of coronary de novo lesions: a comprehensive review. Cardiology and Therapy.

[ref-12] Seedial SM, Ghosh S, Saunders RS, Suwanabol PA, Shi X, Liu B, Kent KC (2013Local). drug delivery to prevent restenosis. Journal of Vascular Surgery.

[ref-13] Stella PR, Belkacemi A, Waksman R, Stahnke S, Torguson R, Strandmann RP.von., Agostoni P, Sangiorgi G (2011). The Valentines Trial: results of the first one week worldwide multicentre enrolment trial, evaluating the real world usage of the second generation DIOR paclitaxel drug eluting balloon for in-stent restenosis treatment. EuroIntervention.

[ref-14] Thygesen K, Alpert JS, Jaffe AS, Simoons ML, Chaitman BR, White HD, Katus HA, Lindahl B, Morrow DA, Clemmensen PM, Johanson P, Hod H, Underwood R, Bax JJ, Bonow RO, Pinto F, Gibbons RJ, Fox KA, Atar D, Newby LK, Galvani M, Hamm CW, Uretsky BF, Steg PG, Wijns W, Bassand JP, Menasché P, Ravkilde J, Ohman EM, Antman EM, Wallentin LC, Armstrong PW, Simoons ML, Januzzi JL, Nieminen MS, Gheorghiade M, Filippatos G, Luepker RV, Fortmann SP, Rosamond WD, Levy D, Wood D, Smith SC, Hu D, Lopez-Sendon JL, Robertson RM, Weaver D, Tendera M, Bove AA, Parkhomenko AN, Vasilieva EJ, Mendis S, Joint ESC/ACCF/AHA/WHF Task Force for the Universal Definition of Myocardial Infarction (2012). Third universal definition of myocardial infarction. Circulation.

[ref-15] Vaquerizo B, Miranda-Guardiola F, Fernández E, Rumoroso JR, Gómez-Hospital JA, Bossa F, Iñiguez A, Oategui I, Serra A (2015). Treatment of small vessel disease with the paclitaxel drug-eluting balloon: 6-month angiographic and 1-year clinical outcomes of the Spanish multicenter registry. Journal of Interventional Cardiology.

[ref-16] Waksman R, Pakala R (2009). Balloon drug-eluting: the comeback kid?. Circulation: Cardiovascular Interventions.

[ref-17] Wöhrle J, Zadura M, Möbius-Winkler S, Leschke M, Opitz C, Ahmed W, Barragan P, Simon JP, Cassel G, Scheller B (2012). SeQuentPlease World Wide Registry: clinical results of SeQuentPlease paclitaxel-coated balloon angioplasty in large-scale, prospective registry study. Journal of the American College of Cardiology.

